# Supplementary material for: Post-Embryonic Lateral Organ Development and Adaxial—Abaxial Polarity Are Regulated by the Combined Effect of ENHANCER OF SHOOT REGENERATION 1 and WUSCHEL in Arabidopsis Shoots
Source: Int J Mol Sci. 2021 Sep 30;22(19):10621. doi: 10.3390/ijms221910621 (PMC8508843; doi:10.3390/ijms221910621)
Supplement: Supplementary file 1 [file ijms-22-10621-s001.zip › Ikeda_etal_Suppl_Figure_legend.pdf]

Figure S1 Analysis of T-DNA insertion position and *ESR1* transcripts in *esr1-2*. (A) The position of T-DNA insertion, name, and primers used for semi-quantitative RT-PCR are described. (B) Semi-quantitative RT-PCR. Primers used for detecting endogenous *ESR1* in 5-day-old Col or *esr1-2* seedlings are described. Number of cycles used for detecting *ESR1* or *TUBULIN3* is 31 or 18, respectively.

Figure S2. The position of T-DNA insertion *phb-101* mutant, SALK_008924C. Primers used for the PCR genotyping are listed in Table S1. Black boxes represent exons and white boxes represent 5' and 3' untranslated regions.

Figure S3. Schematic illustration of expression pattern of STM, WUS, REV, ESR1, and ESR2 proteins in the vegetative shoot. If available, the intensity of protein accumulation is depicted as gradient.
