# Supplementary figures and images for: Post-Embryonic Lateral Organ Development and Adaxial—Abaxial Polarity Are Regulated by the Combined Effect of ENHANCER OF SHOOT REGENERATION 1 and WUSCHEL in Arabidopsis Shoots
Source: Int J Mol Sci. 2021 Sep 30;22(19):10621. doi: 10.3390/ijms221910621 (PMC8508843; doi:10.3390/ijms221910621)

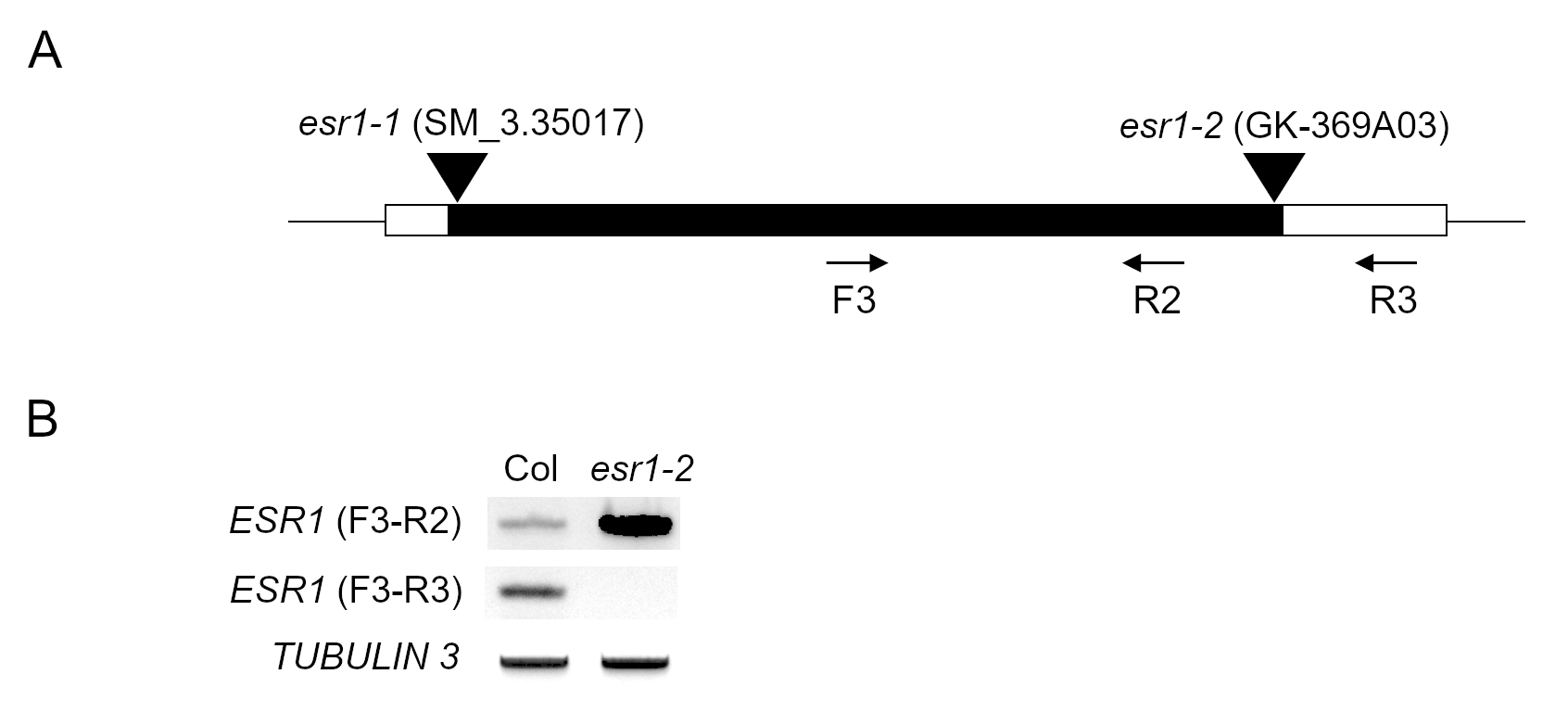

Supplement: Supplementary file 1 [file ijms-22-10621-s001.zip › Figure S1.jpg]

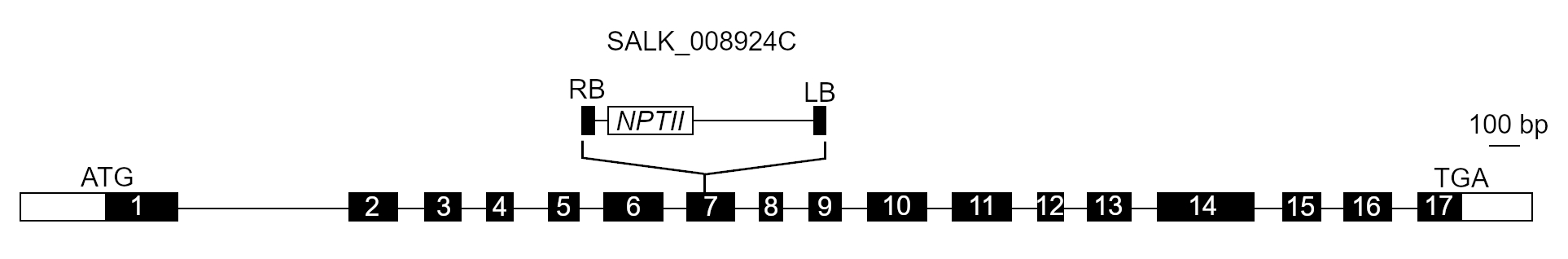

Supplement: Supplementary file 1 [file ijms-22-10621-s001.zip › Figure S2.jpg]

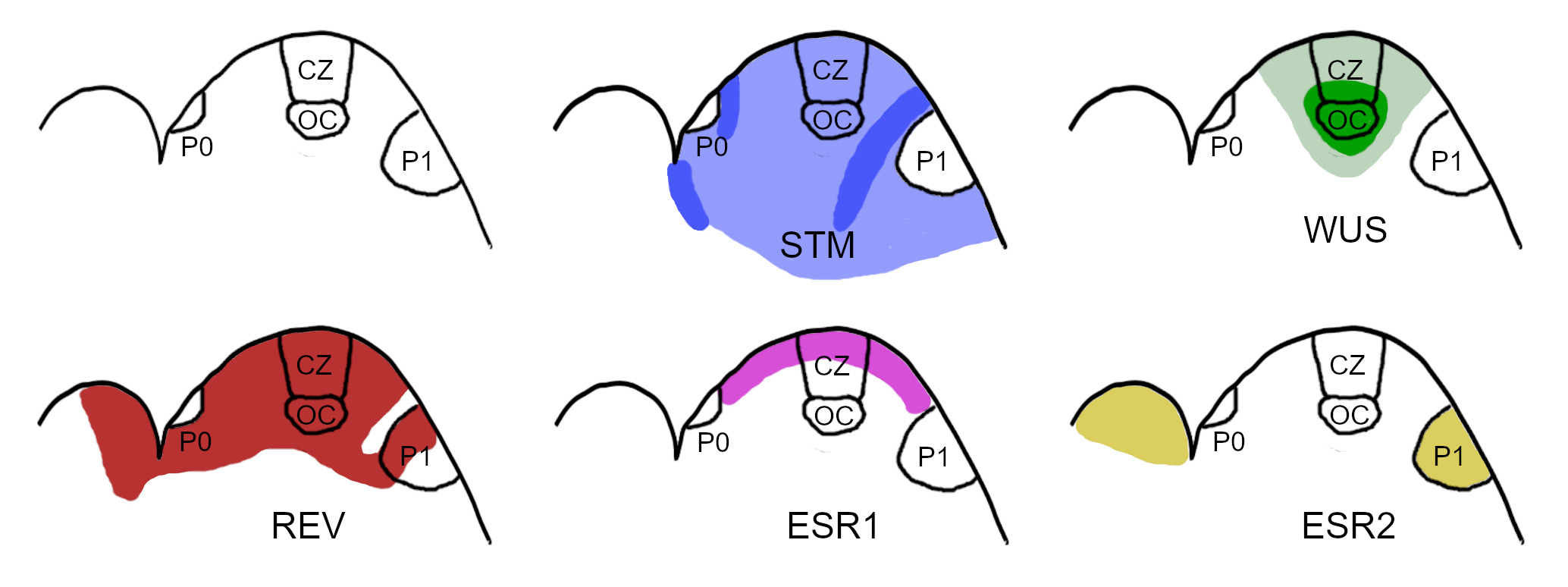

Supplement: Supplementary file 1 [file ijms-22-10621-s001.zip › Figure S3.jpg]
